# Characterization of Mediastinal Bulky Lymphomas with FDG-PET-Based Radiomics and Machine Learning Techniques

**DOI:** 10.3390/cancers15071931

**Published:** 2023-03-23

**Authors:** Elisabetta Maria Abenavoli, Matteo Barbetti, Flavia Linguanti, Francesco Mungai, Luca Nassi, Benedetta Puccini, Ilaria Romano, Benedetta Sordi, Raffaella Santi, Alessandro Passeri, Roberto Sciagrà, Cinzia Talamonti, Angelina Cistaro, Alessandro Maria Vannucchi, Valentina Berti

**Affiliations:** 1 Nuclear Medicine Unit, Department of Experimental and Clinical Biomedical Sciences ‘Mario Serio’, University of Florence, 50139 Florence, Italy; 2 Department of Information Engineering, University of Florence, 50134 Florence, Italy; 3 Istituto Nazionale di Fisica Nucleare (INFN), Florence Division, 50019 Sesto Fiorentino, Italy; 4 Department of Radiology, Azienda Ospedaliero Universitaria Careggi, 50139 Florence, Italy; 5 Hematology Department, Azienda Ospedaliero Universitaria Careggi, University of Florence, 50139 Florence, Italy; 6 Department of Experimental and Clinical Medicine, CRIMM, Center Research and Innovation of Myeloproliferative Neoplasms, Azienda Ospedaliera Universitaria Careggi, University of Florence, 50139 Florence, Italy; 7 Pathology Section, Department of Health Sciences, University of Florence, 50139 Florence, Italy; 8 Medical Physics Unit, Department of Experimental and Clinical Biomedical Sciences ‘Mario Serio’, University of Florence, 50139 Florence, Italy; 9 Nuclear Medicine Department, Salus Alliance Medical, 16128 Genoa, Italy; 10 Pediatric Study Group for Italian Association of Nuclear Medicine (AIMN), 20159 Milan, Italy

**Keywords:** bulky lymphoma, diagnosis, textural analysis, machine learning, FDG-PET, radiomics, precision medicine, personalized treatment

## Abstract

**Simple Summary:**

This manuscript aims to address the diagnostic challenges of mediastinal bulky lymphomas with the baseline value of 18F-FDG PET/CT metabolic, volumetric and texture parameters, also relying on machine learning techniques, in patients with grey zone lymphoma, primary diffuse large B-cell lymphoma of the mediastinum and classical Hodgkin lymphoma. Different types of histology demonstrated several baseline 18F-FDG PET/CT radiomics parameters that were significantly different from one another, suggesting the possibility of identifying potential histological heterogeneity and aggressive transformation. Moreover, using radiomics-based imaging biomarkers, machine learning techniques offer a solution for separating not completely disjoint histological types. To date, the gold standard for diagnosis is biopsy, but machine learning methods could be combined with radiomics to build a histological representation of mediastinal bulky masses that is able to successfully identify different types of lymphomas. Finally, this preliminary study supports the potential of metabolic texture analyses as future imaging biomarkers, with a growing role in clinical diagnosis.

**Abstract:**

Background: This study tested the diagnostic value of 18F-FDG PET/CT (FDG-PET) volumetric and texture parameters in the histological differentiation of mediastinal bulky disease due to classical Hodgkin lymphoma (cHL), primary mediastinal B-cell lymphoma (PMBCL) and grey zone lymphoma (GZL), using machine learning techniques. Methods: We reviewed 80 cHL, 29 PMBCL and 8 GZL adult patients with mediastinal bulky disease and histopathological diagnoses who underwent FDG-PET pre-treatment. Volumetric and radiomic parameters were measured using FDG-PET both for bulky lesions (BL) and for all lesions (AL) using LIFEx software (threshold SUV ≥ 2.5). Binary and multiclass classifications were performed with various machine learning techniques fed by a relevant subset of radiomic features. Results: The analysis showed significant differences between the lymphoma groups in terms of SUVmax, SUVmean, MTV, TLG and several textural features of both first- and second-order grey level. Among machine learning classifiers, the tree-based ensembles achieved the best performance both for binary and multiclass classifications in histological differentiation. Conclusions: Our results support the value of metabolic heterogeneity as an imaging biomarker, and the use of radiomic features for early characterization of mediastinal bulky lymphoma.

## 1. Introduction

The presence of a mediastinal bulky mass at the time of diagnosis in patients with lymphoma is a poor prognostic factor in treatment response and overall survival [1,2,3,4]. Therefore, an accurate characterization of the bulky mass is crucial and represents a diagnostic challenge in clinical and imaging evaluation.

Despite the similarities in clinical presentation, the treatment strategies and outcomes are highly different among the most common mature B-cell lymphomas associated with mediastinal bulky masses, which are classical Hodgkin lymphoma (cHL), primary mediastinal B-cell lymphoma (PMBCL) and grey zone lymphoma (GZL). Since the treatment approaches for cHL and PMBCL are historically different, correctly differentiating their histology is of paramount importance, even if it often represents a non-trivial task. GZL is an uncommon, unclassifiable B-cell lymphoma, with intermediate features between diffuse large B-cell lymphoma and cHL. Furthermore, a spectrum of morphologies with features of cHL and PMBL can occur in GZL. In addition, the same lymphoma type could exhibit not only clinical behavioral differences, but also intra-tumor heterogeneities, reflecting different cell subpopulations distributed in different regions of the same lesion. Both inter- and intra-patient heterogeneity may be found, varying with the histopathological pattern of the lymphoma, resulting in a different prognosis [5].

In this scenario, morphological and functional imaging represents a fundamental diagnostic method to fully characterize bulky mediastinal lymphoma. Nowadays, FDG-PET is considered an indispensable tool for staging and defining the treatment response in different lymphomas. Its value derives both from the intrinsic ability to quantify the use of glucose as a local functional activity, providing information on specific metabolic patterns, and from the continuous refinement of many methodological and interpretative aspects such as metabolic semi-quantification and volumetric parameters as SUV, MTV and TLG [1,2,6,7].

Radiomics is a quantitative approach to medical imaging based on the assumption that biomedical images contain information of disease-specific processes that human eyes cannot detect. It provides several image properties that can be used to characterize tumor phenotypes and support clinical decision-making in a non-invasive manner [8]. Furthermore, texture analysis (TA) quantitively estimates intra-tumor heterogeneity, reflecting the heterogeneities in the distribution of cell subpopulations [9]. Radiomics deeply benefits from the progress achieved by the machine learning (ML) community, relying on state-of-the-art algorithms for medical image segmentation [10,11,12], for the extraction of imaging biomarkers or even for the analysis of textural radiomic signatures [13,14,15]. In the era of precision medicine and personalized treatment [16], metabolic texture analyses may become the future imaging biomarker, with prognostic and predictive values [17].

We assessed the diagnostic value of metabolic FDG-PET and TA in the differentiation of the most common mature B-cell lymphomas associated with mediastinal bulky masses due to cHL, PMBCL and GZL. Then, using radiomic features to feed ML algorithms, we aimed to build a radiomics-based histological representation of mediastinal bulky masses despite the presence of not completely disjoint lymphoma types.

## 2. Materials and Methods

### 2.1. Population

**Figure 1:**
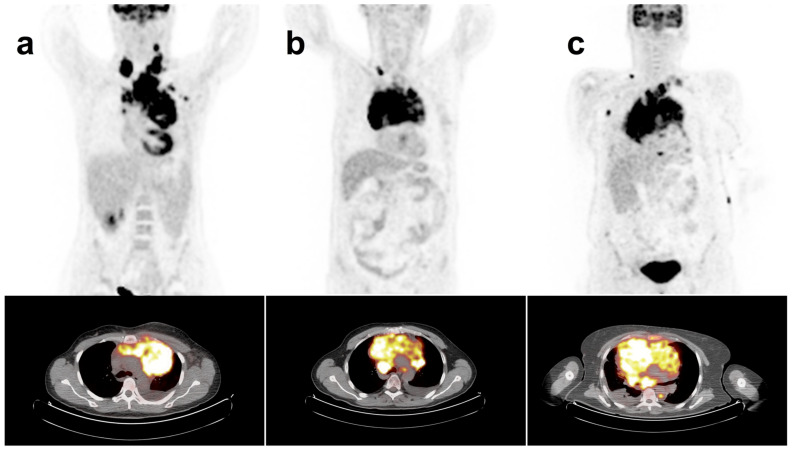
^18^F-FDG-PET/CT maximum intensity projection (MIP) and mediastinal transaxial images show the mediastinal bulky mass in patient with (**a**) cHL, (**b**) GZL and (**c**) PMBCL.

From January 2010 to April 2021, a cohort of untreated patients with a histopathological diagnosis of cHL, PMBCL or GZL were retrospectively evaluated in a monocentric study conducted by both the Nuclear Medicine and Hematology Divisions of Azienda Ospedaliero-Universitaria Careggi (Florence, Italy). Eligibility criteria included ≥18 years, histologically proven cHL, PMBCL and GZL, and the presence of mediastinal bulky disease with diameter ≥5 cm in a computed tomography scan and FDG-PET at baseline (Figure 1). Relapse or refractory patients were excluded from the study. The study was approved by the Institutional Ethics Review Board of the center, and all patients provided written informed consent. The study was conducted according to the Helsinki Declaration, good clinical practice and the applicable national regulations.

### 2.2. Image Acquisition

All patients fasted for at least 4 h before examination, with blood glucose levels < 170 mg/dL. Images from the mid-skull to pelvis were obtained using a dedicated PET/CT scanner (Philips Gemini TF 16 PET/CT), 60 min after intravenous injection of 3.7 MBq/kg of ^18^F-FDG. Before the PET scans, a low-dose CT (120 kV; 50–80 mA) was acquired to allow attenuation correction and lesion localization. PET images were reconstructed using an iterative algorithm (3D LOR RAMLA reconstruction with TOF, FOV: 576, matrix: 144 × 144, voxel dimension: 4 × 4 × 4 mm). Fused images of matching pairs of PET and CT images were available for review in axial, coronal and sagittal planes and in maximum intensity projections (MIPs).

### 2.3. Data Analysis

FDG-PET images were independently evaluated by two nuclear medicine experts, using the open-source software LIFEx 6.3 (www.lifexsoft.org accessed from January 2020 to August 2021) that enables the computation of conventional, histogram-based textural and shape characteristics from medical images [18]. Each measurement was performed using the SUV thresholding method, based on the absolute SUV value > 2.5 on a bulky lesion (BL) [19].

The SUVmax and SUVmean of the BL were also evaluated, computed in SUVbw units for both variables. The segmentation was performed using the default settings suggested by the LIFEx user guide. Regions containing only physiological FDG uptake were edited out. For each bulky lesion, metabolic tumor volume (MTV) and tumor lesion glycolysis (TLG) were computed.

### 2.4. Texture Analysis

The features defined as first-order and second-order were extracted on the BL VOI using LIFEx software [18]. First-order features are typically drawn from the histogram of grey-level values obtained from the considered VOIs. They describe the overall signal variation in the VOI regardless of the relative position of the voxels. Therefore, these features are invariant to geometric transformations, and robust to image reconstruction and filtering. They include basic statistics such as mean, median, range, standard deviation, skewness and kurtosis.

Second-order features are those that are usually referred to as *texture features*, since they consider the spatial relationship between neighboring VOIs in an image; thus, they are capable of capturing details on lesion heterogeneity. They include the grey-level co-occurrence matrix (GLCM), the neighborhood grey-level different matrix (NGLDM), the grey-level run-length matrix (GLRLM) and the grey-level zone-length matrix (GLZLM), and indices from sphericity and histogram. A detailed description of the various texture parameters provided by LIFEx can be found at www.lifexsoft.org accessed from January 2020 to August 2021.

The conventional and advanced metabolic tumor parameters as well as the radiomics features assessed are summarized in Table 1.

### 2.5. Machine Learning

GZL exhibits mixed characteristics between cHL and PMBCL. This represents a major difficulty in the differentiation of the three mediastinal lymphoma types. Particularly, it prevents the direct performing of multiclass classifications. To build a representation of mediastinal lymphomas that considers this limitation, the ML study was divided into two steps:

- Training a model to perform a binary classification between cHL and PMBCL;
- Promoting the trained model to cope with a multiclass classification.

Since it was a preliminary study, several ML strategies were compared in order to assess their capability to build a reliable histological representation for the three lymphoma types. The performance of logistic regression, support vector machine (with linear kernel), Gaussian process, random forest and gradient-boosted decision trees (BDT) are discussed in the following sections of this document.

The ML classifiers were trained on a sample of the study cohort containing 80% of patients, namely the training set. The model training was driven by an independent and robust subset of the radiomic features extracted from LIFEx [18]. Feature importance was measured with the recursive feature elimination (RFE) algorithm [20]. When possible, hyperparameter optimization (HPO) was used to enhance the discriminant capabilities of binary classifiers. In particular, HPO studies were carried out using Bayesian optimization methods [21,22] to maximize the AUC score obtained by tree-based ensemble models in an independent subset of the training set (validation set).

The performance of both binary and multiclass classifiers was evaluated with the AUC score computed on a separate sample containing 20% of never-seen patients, namely the test set. Given the limited statistic of the study, preventing models from overtraining and controlling possible bias is crucial. Thus, the robustness of the trained classifiers and the uncertainties associated with their performance were measured through the bootstrap technique. The various ML models were implemented in Python (the code for our models is available at https://github.com/mbarbetti/mediastinal-lymphoma-classification) using the Scikit-Learn package [23], while the Bayesian optimization of the tree-based ensembles was driven by the Optuna framework [24].

Following what was undertaken by Montes de Jesus et al. [25], the performance achieved by the trained classifiers was compared to what was obtained using the SUVmax parameter alone, which was used as a baseline for the benchmark studies.

**Figure 2:**
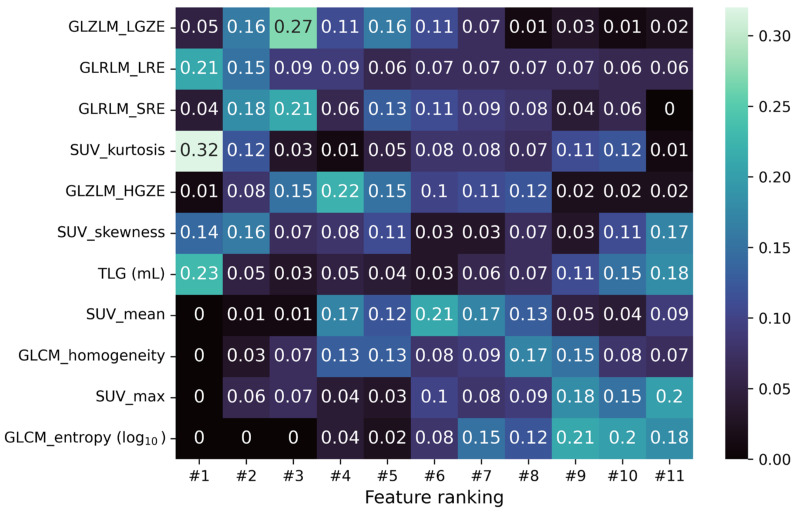
Matrix $N_{\mathrm{features}}\times N_{\mathrm{features}}$ reporting the fraction of times the *i*-feature ends up in the *j*-position of the ranking obtained from the RFE algorithm during a 100-iterated bootstrap technique on the logistic regression training.

Before training any model, an intense data cleaning was performed, keeping only a limited number of the radiomic features extracted from LIFEx [18]. The intensity-based features were selected to ensure highly independent parameters (|PCC| > 0.75) and to provide the SUVmax value. The texture features were taken in line with Orlhac et al. [26], selecting the indices robust to segmentation and relatively independent from one another. Lastly, to test the discriminant power of each feature, a Mann-Whitney U test [27] was performed on the chosen subset, requiring a confidence level of 99% to reject the null hypothesis. A complete list of the selected features is reported in Figure 2 together with a probabilistic estimation of the relative ranking computed after 100 iterations of the RFE algorithm on the logistic regression training.

### 2.6. Statistical Analysis

Statistical analysis was performed with SPSS software (SPSS25) by means of ANOVA to evaluate the differences between means among measured variables. Quantitative data are presented as the mean  ±  standard deviation (SD). *p* value < 0.05 was considered significant.

## 3. Results

### 3.1. Patients

One-hundred-seventeen histologically proven patients were included in the study (Table 2). Twenty-nine patients were PMBCL (24.8%), 80 (68.4%) were cHL, and 8 (6.8%) were GZL. The median age for PMBCL was 40 years (range, 21–59), for cHL it was 33 years (range, 18–74) in the 73 available patients, and for GZL it was 47 years (range, 18–60). The median largest mediastinal diameter measured for PMBCL was 11.0 cm (range, 6.5–17), 8.9 cm (range, 5–20) in cHL patients and 13.5 cm (range, 5–16) in GZL patients.

### 3.2. Radiomic Features

**Figure 3:**
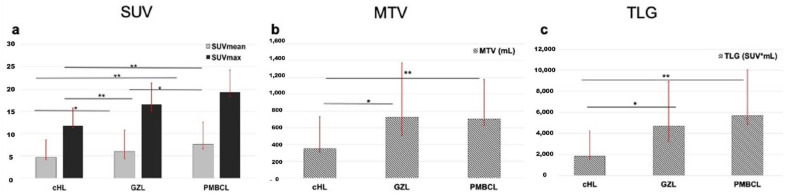
Histograms representing mean SUVmax and SUVmean (**a**), MTV (**b**) and TLG (**c**) in the subsets of cHL, GZL and PMBCL. * p < 0.05, ** *p* < 0.01.

Several metabolic and volumetric indices, as well as texture features, were significantly different among the three groups. SUVmax and TLG were significantly different between groups, with the highest values in PMBCL, intermediate in GZL and lowest in cHL (Figure 3). PMBCL and GZL had similar MTV values, but they were significantly higher than cHL.

Among first-order variables, histogram skewness and kurtosis were significantly different between groups (*p* < 0.001). PMBCL showed significantly lower values with respect to the other groups, indicating asymmetrical histograms with left-sided skewness. On the contrary, cHL showed significantly higher positive values than the other groups, indicating asymmetrical histograms with right-sided skewness.

Regarding kurtosis, cHL showed significantly higher kurtosis values, indicating a value distribution with a heavier tail as compared to PMBCL, which showed significantly lower kurtosis values.

Among the features extracted from NGLDM, only contrast resulted significantly different between groups, with PMBCL showing significantly higher contrast than GZL and cHL, and GZL significantly higher than cHL.

**Figure 4:**
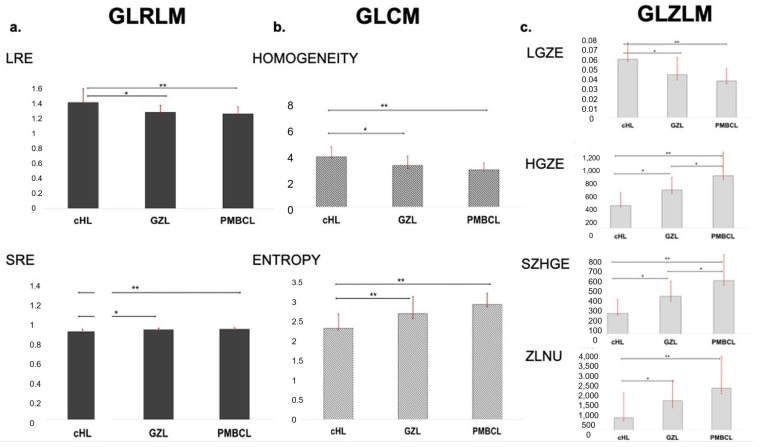
Histograms representing mean LRE and SRE derived from GLRLM (**a**), homogeneity and entropy derived from GLCM (**b**) and LGZE, HGZE, SZHGE and ZLNU derived from GLZLM (**c**) in the subsets of cHL, GZL and PMBCL. * *p* < 0.05, ** *p* < 0.01.

In the GLZLM matrix, GLZLM_SZE, GLZLM_HGZE, GLZLM_SZHE and GLZLM_ZLNU parameters showed significantly increasing values between groups, with lower values in cHL, intermediate values in GZ and higher values in PMBCL. On the contrary, GLZLM_LZE, GLZLM_LGZE and GLZLM_SZLGE parameters showed significantly decreasing values between groups, with significantly higher values in cHL, as compared to both GZL and PMBCL (Figure 4).

GLCM features showed significantly different results between groups: variance, entropy and dissimilarity showed lower values in cHL, higher values in PMBCL and intermediate values in GZL. On the contrary, GLCM homogeneity resulted significantly lower in cHL, intermediate in GZL and higher in PMBCL.

Among the GLRLM features, GLRLM_LRE, GLRLM_LRLGE, GLRLM_SRLGE, GLRLM_HGRE and GLRLM_RP showed significantly lower values in cHL, intermediate values in GZL and higher values in PMBCL. Instead, in GLRLM_ SRHGE and GLRLM_ SRE, higher values were seen in cHL, with intermediate values in GZL and lower values in PMBCL. Regarding GLRLM_RLNU, PMBCL and GZL showed similar values, both significantly higher than cHL.

### 3.3. Machine Learning

**Figure 5:**
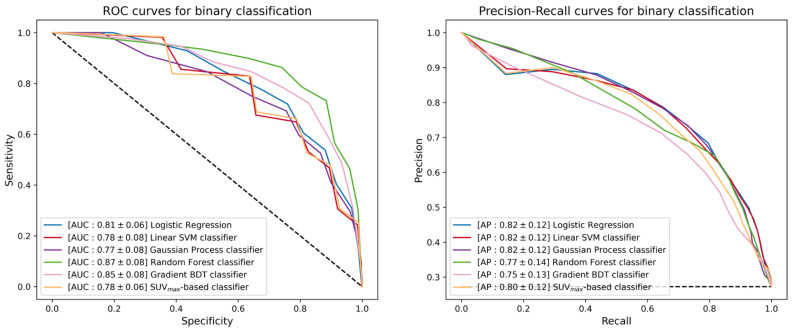
ROC curves (**left**) and precision-recall curves (**right**) of the different binary classifiers under investigation. Each classifier is reported together with mean and standard deviation (in square brackets) of the AUC and AP scores achieved during a 300-iterated bootstrap technique.

The use of ML techniques assumes that the training set is statistically significant, not only in terms of instance multiplicity but also in terms of the discriminant power brought by each instance. The first step of the ML study was the separation between cHL and PMBCL. Since the patient cohort dataset was highly unbalanced, the SMOTE technique [28] was applied to the training set to boost the lower-represented class through over-sampling and to prevent models from overtraining. The five classifiers were trained over such SMOTE-corrected sample, while their performance was evaluated using the unbalanced test set. The true positive rate (TPR, also known as sensitivity or recall), true negative rate (TNR, also called specificity), positive predictive value (PPV, also referred to as precision), AUC-ROC (AUC) and AUC-PR (AP) scores of different classifiers are listed in Table 3. Figure 5 reports the ROC curves (left) and the precision-recall curves (right), together with the corresponding AUC and AP scores. The TPR and TNR scores were computed using two custom decision thresholds chosen to require strict performance in output to the classifiers; in particular, the scores reported in Table 3 correspond to a TPR score greater than 0.7 and 0.9 on the training set. The random forest classifier achieved the best performance with an AUC score of 0.87 against the 0.78 score achieved by the SUVmax-based classifier.

**Figure 6:**
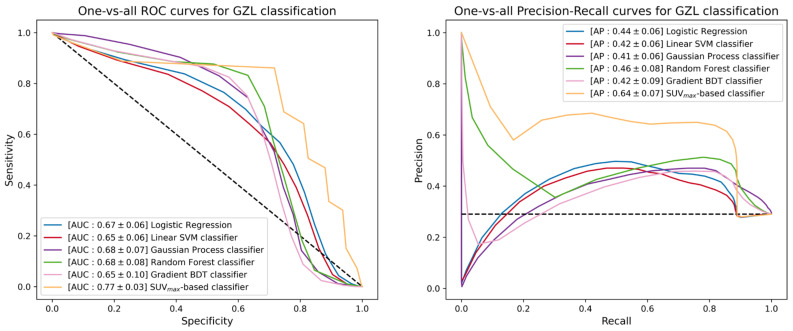
ROC curves (**left**) and precision-recall curves (**right**) of the different multiclass-promoted binary classifiers under investigation. Each classifier is reported together with mean and standard deviation (in square brackets) of the one-vs-all AUC and AP scores achieved during a 300-iterated bootstrap technique for GZL classification.

**Figure 7:**
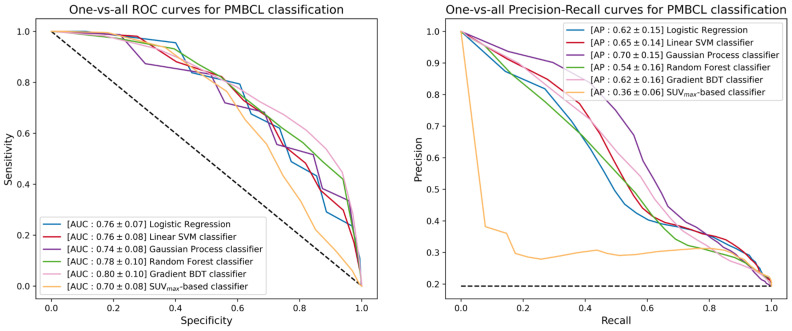
ROC curves (**left**) and precision-recall curves (**right**) of the different multiclass-promoted binary classifiers under investigation. Each classifier is reported together with mean and standard deviation (in square brackets) of the one-vs-all AUC and AP scores achieved during a 300-iterated bootstrap technique for PMBCL classification.

The next step of the ML study was the promotion of the five binary classifiers to include GZL within the available classes. Since GZL can be described as a sort of classification uncertainty between cHL and PMBCL, its representation can be implemented by cutting out a decision boundary in the space induced by the trained models between the regions depicting the two separate classes. The one-vs-all TPR, TNR, PPV, AUC and AP scores for GZL and PMBCL of promoted classifiers are listed in Table 4. Figure 6 reports the one-vs-all ROC curves (left) and precision-recall curves (right), together with the corresponding AUC and AP scores for the GZL classification. Similar curves and scores are depicted in Figure 7 for the PMBCL classification. Again, the random forest classifier achieved the best performance with one-vs-all AUC scores of (0.68, 0.78) against the (0.77, 0.70) totaled by the SUVmax-based classifier.

## 4. Discussion

This study showed the diagnostic value of FDG-PET volumetric and texture parameters in discriminating mediastinal bulky lymphomas caused by different histological types, i.e., GZL, PMBCL and cHL.

Among conventional quantitative variables, SUV measurements showed significantly different results between the three lymphoma types, with lower values in cHL, intermediate in GZL and higher in PMBCL.

SUVs have been the first and most investigated measures to provide objective quantification of tumor biological activity. Several studies in lymphoma patients correlated FDG activity with tumor proliferative ability, demonstrating that high-proliferative lymphomas, such as PMBCL, often show very high metabolic activity [29,30,31]. Our results are consistent with these findings, confirming that differences in proliferation activity may be a possible explanation for the different FDG uptakes between various histo-types. Moreover, both SUVmax and SUVmean values were significantly lower in cHL compared with NHL, consistent with its peculiar neoplastic tissue architecture, composed of few and scattered neoplastic cells called Hodgkin and Reed-Sternberg cells, accounting for less than 1% of the total cell count, surrounded by an overwhelming population of non-neoplastic mononuclear bystander cells with relatively high metabolism [32,33,34,35]. Such unique pathological features may explain the differences in metabolic activity of cHL compared to PMBCL. Finally, we found an intermediate behavior in the GZL group, again consistent with its biological characteristics. Indeed, GZL could show a wide range of cell types, with the coexistence of cells resembling HRS cells, cells with centroblastic or immunoblastic cytology and marked nuclear pleomorphism, and cells with more monomorphic cytology, resembling PMBCL [36].

Beyond SUV values, there were also significant differences among lymphoma types regarding volume-based variables, such as MTV and TLG. MTV represents one important prognostic factor in lymphoma, in addition to stage and the other clinical factors such as LDH, performance status, age and presence of extra-nodal disease. Not surprisingly, most studies have demonstrated that MTV was an adverse prognostic factor, regardless of lymphoma histology and choice of quantification method [37,38,39,40].

Our data are in line with the concept that volumetric parameters have greater values in aggressive histo-types. Indeed, in our study, PMBCL and GZL showed higher values of both MTV and TLG than cHL, but at the same time PMBCL significantly differs from GZL due to the higher value of TLG.

One of the most interesting findings is the significant difference between lymphoma types regarding several texture features extracted from FDG-PET images. Metabolic heterogeneity is a remarkable feature of malignancy, and texture analysis provides tools for its quantification [41]. In recent years, several studies convincingly showed the complex relationship between textural features and biological characteristics of a tissue and how the spatial intensity variations found in an image could differ depending on biological processes. This association has been found to be more robust for high-order features, which refer to the relative distribution of intensity value within the neighborhood, than first-order variables, which only measure intensity variability independently of the underlying spatial distribution in the tumor microenvironment. Those features measuring increasing heterogeneity within tumors may be associated with differences in regional tumor cellularity, proliferation, hypoxia, angiogenesis and necrosis, but also genomic variety, factors that independently have been associated with more aggressive behavior, poorer response to treatment and worse prognosis [42,43,44]. We chose to focus on patients presenting with mediastinal bulky lymphomas since it is well established that the complementary information provided by textural analysis strongly depends on tumor size. Although information increases substantially with larger volumes, the level of correlation between the variables tends to decrease substantially [36,45].

In our study, the high co-occurrence features, analyzing inter-relationships between pairs of voxels and thus characterizing local non-uniformities, were able to significantly differentiate the three lymphoma groups. Orlhac et al. [46] reported that low grey-level zone emphasis exhibited higher values in visually homogeneous lesions than in heterogeneous lesions. They identified two different sets of texture indices able to reflect the different characteristics of uptake heterogeneity. The first included GLCM_homogeneity, GLCM_entropy, GLRLM_SRE and GLRLM_LRE, all of which were sensitive to the presence of uptake heterogeneity, in absence of the influence of the nature of the heterogeneity (hyposignal or hypersignal). The second comprised GLZLM_HZE and GLZLM_LZE, which were mostly sensitive to the average uptake rather than to the local heterogeneity uptake. So, homogeneous lesions like cHL had a higher value of homogeneity and GLZM_LGZE and a lower value of entropy and GLZM_HGZE than heterogeneous lesions such as PMBCL and GZL. As expected, entropy, which reflects disorder, varies in the opposite direction to homogeneity. Another important aspect is how SUV measurements and entropy are strongly correlated [47,48]. Since the more chaotic the image, the greater the entropy will be, this is reflected in the trend of our results, too, with higher values in PMBCL and lower values in cHL [8,49].

The other features evaluated in this study highlight tumor heterogeneity, depending on the type of matrix used and the kind of feature computed on this matrix. Consequently, whereas a single feature cannot be directly linked to a specific biological process, one could assume that a combination of textural parameters may be closely related to underlying pathophysiological processes, such as vascularization, neoangiogenesis, tumor aggressiveness or hypoxia [50,51].

Finally, referring to the GLZM matrix, the “Zone-Length Non-Uniformity”, which measures the variability of the intensity values of the grey-level in the image, showed lower values, indicating greater homogeneity in the intensity values as in the cHL and instead very high values in the PMBCL.

The literature studying the possibility of pathological predictions of histological subtypes based on radiomic features from FDG-PET images has produced conflicting results. Previous studies excluded the possibility of creating a close correlation between histology and radiomics, even if texture indices may provide some useful information in the spatial organization of tumor cells [52]. However, more recently, ML has been shown to provide promising methods for predicting the different subtypes of malignant lymphomas based on radiomics [26,53].

Our data are consistent with these latter results, demonstrating that ML algorithms powered by radiomics and texture features can build robust histological-based classifiers, with good discriminant power. In fact, through a set of selected features (reported in Figure 2), the ML algorithms show promising results in inferring the histological type of the mediastinal bulky masses evaluated with a good sensitivity and specificity for the binary classification PMBCL/cHL, as well as with acceptable performance for the multiclass classification.

Among the set of radiomic and texture features selected for model training, there is no strong indication of the importance order of such features in the classification task. What appears in Figure 2 on the feature ranking obtained by iterating 100 times the RFE algorithm on the logistic regression training is that SUV kurtosis is the best ranked feature most of the time. The texture GLRLM and GLZLM indices follow, achieving high-ranked positions on average. On the contrary, GLCM_entropy and SUVmax seem to be less important for the logistic regression peaking at low-ranked positions. SUVmean and GLCM_homogeneity look to be moderately important, while it is hard to infer something about the importance of TLG and SUV skewness from Figure 2.

The best performing binary classifier is the random forest that, with an AUC score of 0.87, totalized a balanced set of scores in TPR and TNR. Referring to the decision threshold with TPR > 0.9 (on the training set), the random forest can correctly classify PMBCL in 81% of patients, and cHL in 80%. The other ML models achieve slightly better performance in identifying PMBCL cases (82–85%), at the cost of lower performing capabilities for cHL identification (77–79%). The SUVmax-based classifier exacerbates this behavior, totalizing the best sensitivity score (88%), but the worst specificity score (63%).

The multiclass problem confirms the domain of the random forest classifier as the best performing model with one-vs-all AUC scores for GZL/PMBCL classification of 0.68/0.78. The ensemble classifier can identify PMBCL lymphomas within a multi-type cohort in 80% of patients, and GZL cases in 86%, keeping acceptable performance in correctly excluding GZL/PMBCL instances for at least 56% of patients. The other ML models show similar multiclass capabilities, even if there are some cases where higher scores in TPR are at the cost of worse performance in TNR and vice versa (Table 4). Again, the SUVmax-based classifier exhibits the same behavior, obtaining the best TPR scores (88% for PMBCL and 89% for GZL), but failing in correctly excluding GZL/PMBCL instances in 55% of all cases.

Despite the fact that SUVmax delivers information about the histological nature of masses that can be tuned to identify specific lymphoma types, such information is not enough to finalize the complementary task that is shown with a significant drop in the specificity performance. Including more features besides the SUV-based ones makes it possible to recover the missing information, providing more balanced performance. Our results on the conjunction of radiomics with ML methods demonstrates that textural features derived from FDG-PET imaging are correlated with histologic diagnosis in patients with mediastinal bulky lymphomas, and thus may support pathological analyses.

In this context, FDG-PET could aim to be used in several additional ways beyond the standard clinical use. On one hand, it could represent the best method for guiding a biopsy in a bulky mass by answering the questions “where and how many samples”, identifying the metabolic patterns corresponding to the specific histology as well. On the other hand, FDG-PET radiomics could help clarify possible discordances between the histopathological diagnosis and clinical characteristics of the patient. In this last case, radiomic features of the bulky mass could represent an additional variable that could help clinicians and pathologists define the diagnosis or guide the decision to undergo a second biopsy in a more suggestive area of the bulky lesion.

The literature on functional imaging radiomics for lymphoma remains scant. Nevertheless, most of the published researchers in this field highlight the capacity of PET-related ML to differentiate lymphomas from other entities [54,55]. Just recently, Montes de Jesus et al. [25] analyzed ML algorithms to discriminate follicular lymphoma from diffuse large B-cell lymphoma with promising results.

The present study had some limitations. Firstly, this was a retrospective study. Secondly, the PMBCL and GZL cohorts were limited, especially compared to the cHL group, even if such proportions reflect the true incidence of these diseases. Moreover, it should be highlighted that most of the results come with large uncertainties, surely due to the limited statistic available. Thus, it could be important to extend this research in a multicenter retrospective study with an enlarged sample size to develop more robust histological-based classifiers and to validate the results in a prospective multicenter one. To harmonize radiomic features calculated from images coming from different scanners or protocols, the use of harmonization methods is of key importance. Moreover, with the current technological evolution, it may also be interesting to implement our approach in full digital PET/CT to confirm our findings. Finally, the relationship between texture parameters and histopathological findings also requires further study to determine the tumor biological dependency of each textural feature.

## 5. Conclusions

Different subtypes of bulky mediastinal lymphomas, namely cHL, GZL and PMBCL, demonstrated different FDG-PET characteristics and metabolic heterogeneity when examined using radiomics and texture analysis. ML methods could be combined with radiomics to build a histological representation of bulky mediastinal masses that can successfully identify different types of lymphomas. In the era of precision medicine and personalized treatment, this preliminary study supports the potential of metabolic texture analyses as a future imaging biomarker, with a growing role in clinical diagnosis.

## Figures and Tables

**Table 1:** Parameters evaluated included conventional and volumetric indices and texture features.

Index Matrix Parameter	Matrix	Parameter
Conventional indices		SUVmean, SUVmax, SUVpeak
Volumetric indices		MTV, TLG
Texture features: first-order	Histogram	skewness, kurtosis, entropy, energy
Texture features: second-order	GLCM	homogeneity, energy, contrast, correlation, entropy, dissimilarity
	NGLDM	coarseness, contrast, busyness
	GLRLM	SRE/LRE, LGRE/HGRE, SRLGE/SRHGE, LRLGE/LRHGE, GLNUr/RLNU, RP
	GLZLM	SZE, LZE, LGZE, HGZE, SZLGE, SZHGE, LZLGE, LZHGE, GLNUz, ZLNU, ZP

**Table 2:** Summary of the study population.

Characteristics	PMBCL, *n* (%)	cHL, *n* (%)	GZL, *n* (%)
Patients	29 (24.8%)	80 (68.4%)	8 (6.8%)
Male sex	10 (34.5%)	35 (43.8%)	5 (62.5%)
Median age y (range)	40 (21–59)	33 (18–74)	47 (16–60).
Ann Arbor Stage			
I	6 (21%)	1 (2%)	0 (0%)
II	12 (58%)	48 (66%)	7 (87.5%)
III	2 (7%)	12 (16%)	1 (12.5%)
IV	4 (14%)	12 (16%)	0 (0%)
B symptoms	8 (28%)	29 (40%)	4 (50%)
LDH UI/l (range)	355 (134–757)	207 (135–630)	255.5 (206–335)
ERS mm/h (range)	37 (6–106)	321 (4–120)	34.5 (20–62)
Median bulky diameter cm (range)	11.0 (6.5–17)	8.9 (5–20)	13.5 (5–20)

**Table 3:** Performance of different machine learning models and the SUVmax-based classifier in separating PMBCL and cHL lymphomas. TPR, TNR and PPV scores are reported for two user-defined decision thresholds, indicated in the table as tight/slight requirements and obtained requiring a sensitivity score greater than 0.9/0.7 on the training set.

		Logistic Regression	Linear SVM	Gaussian Process	Random Forest	Gradient BDT	${\mathrm{SUV}}_{\max}$
AUC [10th, 32nd percentiles]		0.81 [0.73, 0.78]	0.78 [0.68, 0.75]	0.77 [0.68, 0.75]	0.87 [0.79, 0.83]	0.85 [0.74, 0.81]	0.78 [0.73, 0.75]
AP [10th, 32nd percentiles]		0.82 [0.64, 0.77]	0.82 [0.65, 0.76]	0.82 [0.66, 0.78]	0.77 [0.56, 0.69]	0.75 [0.58, 0.69]	0.80 [0.63, 0.75]
TPR [10th, 32nd percentiles]	Tight requirement	0.84 [0.67, 0.83]	0.82 [0.67, 0.83]	0.85 [0.67, 0.83]	0.81 [0.50, 0.67]	0.59 [0.33, 0.50]	0.88 [0.67, 0.83]
	Slight requirement	0.69 [0.50, 0.67]	0.69 [0.33, 0.67]	0.69 [0.50, 0.67]	0.65 [0.33, 0.50]	0.62 [0.33, 0.50]	0.76 [0.50, 0.67]
TNR [10th, 32nd percentiles]	Tight requirement	0.79 [0.62, 0.75]	0.79 [0.62, 0.75]	0.77 [0.56, 0.75]	0.80 [0.62, 0.75]	0.90 [0.81, 0.88]	0.63 [0.38, 0.56]
	Slight requirement	0.90 [0.81, 0.88]	0.90 [0.81, 0.88]	0.91 [0.81, 0.88]	0.91 [0.81, 0.88]	0.91 [0.81, 0.88]	0.82 [0.62, 0.75]
PPV [10th, 32nd percentiles]	Tight requirement	0.13 [0.11, 0.12]	0.14 [0.11, 0.12]	0.14 [0.12, 0.13]	0.15 [0.10, 0.14]	0.16 [0.09, 0.14]	0.12 [0.10, 0.11]
	Slight requirement	0.18 [0.13, 0.16]	0.18 [0.10, 0.17]	0.18 [0.13, 0.17]	0.17 [0.09, 0.14]	0.17 [0.09, 0.14]	0.18 [0.14, 0.16]

**Table 4:** Performance of different machine learning models and the SUVmax-based classifier in separating cHL, GZL and PMBCL lymphomas. One-vs-all TPR, TNR and PPV scores are reported imposing the tight requirement, namely requiring the TPR score of the binary classifier greater than 0.9 on the training set.

		Logistic Regression	Linear SVM	Gaussian Process	Random Forest	Gradient BDT	${\mathrm{SUV}}_{\max}$
AUC [10th, 32nd percentiles]	One-vs-all for GZL classification	0.67 [0.60, 0.65]	0.65 [0.58, 0.63]	0.68 [0.58, 0.65]	0.68 [0.59, 0.66]	0.65 [0.52, 0.62]	0.77 [0.73, 0.77]
	One-vs-all for PMBCL classification	0.76 [0.66, 0.72]	0.76 [0.66, 0.72]	0.74 [0.63, 0.71]	0.78 [0.65, 0.74]	0.80 [0.66, 0.76]	0.70 [0.59, 0.67]
AP [10th, 32nd percentiles]	One-vs-all for GZL classification	0.44 [0.37, 0.41]	0.42 [0.35, 0.39]	0.41 [0.33, 0.38]	0.46 [0.37, 0.42]	0.42 [0.31, 0.38]	0.64 [0.55, 0.60]
	One-vs-all for PMBCL classification	0.62 [0.42, 0.56]	0.65 [0.46, 0.58]	0.70 [0.50, 0.63]	0.54 [0.33, 0.45]	0.62 [0.40, 0.55]	0.36 [0.28, 0.33]
TPR [10th, 32nd percentiles]	One-vs-all for GZL classification	0.74 [0.67, 0.67]	0.67 [0.56, 0.67]	0.86 [0.78, 0.89]	0.86 [0.89, 0.89]	0.66 [0.22, 0.56]	0.89 [0.89, 0.89]
	One-vs-all for PMBCL classification	0.84 [0.67, 0.83]	0.81 [0.65, 0.67]	0.85 [0.67, 0.83]	0.80 [0.50, 0.67]	0.76 [0.50, 0.67]	0.88 [0.67, 0.83]
TNR [10th, 32nd percentiles]	One-vs-all for GZL classification	0.60 [0.41, 0.55]	0.63 [0.45, 0.59]	0.59 [0.41, 0.55]	0.64 [0.45, 0.59]	0.66 [0.50, 0.64]	0.49 [0.27, 0.41]
	One-vs-all for PMBCL classification	0.59 [0.44, 0.67]	0.63 [0.52, 0.60]	0.53 [0.40, 0.52]	0.56 [0.44, 0.52]	0.64 [0.48, 0.60]	0.45 [0.28, 0.40]
PPV [10th, 32nd percentiles]	One-vs-all for GZL classification	0.44 [0.35, 0.40]	0.43 [0.35, 0.40]	0.47 [0.38, 0.44]	0.50 [0.38, 0.47]	0.44 [0.26, 0.40]	0.43 [0.27, 0.41]
	One-vs-all for PMBCL classification	0.33 [0.27, 0.31]	0.35 [0.28, 0.32]	0.31 [0.25, 0.29]	0.31 [0.24, 0.29]	0.35 [0.25, 0.30]	0.28 [0.22, 0.25]

## Data Availability

Anonymized data for our analyses presented in this report are available upon request from the corresponding authors.
